# Neuroimaging, nutrition, and iron-related genes

**DOI:** 10.1007/s00018-013-1369-2

**Published:** 2013-07-02

**Authors:** Neda Jahanshad, Priya Rajagopalan, Paul M. Thompson

**Affiliations:** Imaging Genetics Center, Laboratory of Neuro Imaging, Department of Neurology, UCLA School of Medicine, Neuroscience Research Building 225E, 635 Charles Young Drive, Los Angeles, CA 90095-1769 USA

**Keywords:** Neuroimaging, Genetics, Iron, Diet, Transferrin, *HFE*, Nutrition, Brain development

## Abstract

Several dietary factors and their genetic modifiers play a role in neurological disease and affect the human brain. The structural and functional integrity of the living brain can be assessed using neuroimaging, enabling large-scale epidemiological studies to identify factors that help or harm the brain. Iron is one nutritional factor that comes entirely from our diet, and its storage and transport in the body are under strong genetic control. In this review, we discuss how neuroimaging can help to identify associations between brain integrity, genetic variations, and dietary factors such as iron. We also review iron’s essential role in cognition, and we note some challenges and confounds involved in interpreting links between diet and brain health. Finally, we outline some recent discoveries regarding the genetics of iron and its effects on the brain, suggesting the promise of neuroimaging in revealing how dietary factors affect the brain.

## Introduction

The human brain changes dynamically throughout life, and profound changes occur from childhood to old age, and, in particular, with the progression of disease. The structural integrity of the living brain may be evaluated with brain scans obtained through a range of neuroimaging techniques. These brain scans include, among others, high-resolution magnetic resonance imaging (MRI) and diffusion-based MRI. Standard anatomical MRI has been the mainstay of clinical neuroradiology for over two decades, and has helped reveal signs of brain aging, such as cortical atrophy, vascular changes, and changes over time in the gray and white matter. More recently, “diffusion-based” MRI has been embraced by neuroscientists and clinical researchers alike, as it can assess microstructural properties of the white matter fibers and the breadth of their connections. This technology is offering new insights into how the brain is organized, how it changes as we age, and what factors affect the changing brain.

Clearly, a wide range of neuroanatomical variation is expected in patients diagnosed with neurological or psychiatric illness. However, even in groups of healthy individuals, brain structure and integrity vary widely. For many measures of human brain morphometry and integrity, around half of the normal variance is due to the genetic variations among us. Even so, environmental factors also affect the brain—both structurally and functionally. Among these factors are education, diet, and stress.

One of the most basic—and to some extent controllable—influences on brain structure and function is a person’s diet and nutritional intake. In addition to proteins (and certain fats, vitamins, and other important minerals such as zinc, copper, iodine, selenium), iron is a nutrient with profound effects on the brain throughout life [1]. Iron’s effects on the brain have been a topic of interest for many years. Iron is critical for healthy brain development, and abnormal iron accumulation in the brain can promote neurodegenerative diseases [2]. In the past few decades, medical and public opinion has changed from favoring iron supplementation in healthy people as they age, to being more circumspect about its potential negative consequences [3].

Many nutrients, including iron, are not metabolized, transported, or processed identically in all individuals. They are regulated by a variety of genes, proteins, and their interactions. In most cases, iron enters the body solely through our diet, and exists as heme and non-heme iron in the body. Specific genes and regulatory proteins are involved in iron metabolism and are responsible for storing iron locally (ferritin) and transporting it (transferrin) throughout the body. The uptake and storage of iron in the brain differs across the brain structures and the distribution patterns also change with aging. It is perhaps not surprising then that genes involved in iron processing and transport are critical in shaping the human brain. However, the specific mechanisms of these effects are only just beginning to be understood.

## Genetic diversity and the brain

In recent years, the field of “imaging genetics” [4] has matured significantly. Imaging genetics aims to correlate brain imaging measures from large groups of people to commonly carried variants in their DNA, in an effort to understand how genetic variation affects the brain and our risk for disease. Initial studies have offered a deeper understanding of how “disease risk” genes may affect the brain, and how they affect brain function and behavior [5].

Carriers of certain common genetic variants—or single-nucleotide polymorphisms (SNPs)—tend to have characteristic differences in brain structure that can be identified, at least at the group level, on MRI scans. These differences in brain integrity suggest how these genes may act to change neural circuitry and neural pathways, affecting a person’s aggregate risk for diseases such as Alzheimer’s disease, schizophrenia, and even autism. Some genetic variants are very common—and have substantial effects on the brain. These include commonly carried variants in the Alzheimer’s disease risk genes, *ApoE* [6], *CLU* [7], and *MTHFR* [8]. Brain variations have also been associated with carrying risk genes for psychiatric illness such as *DISC1* [9] and neuregulin [10, 11], and risk genes for developmental disorders such as autism, including *CNTNAP2* and *MET* [12, 13], among others. The flood of brain imaging and genetic data, available now on a large scale, has also made it possible to search the entire genome to identify new genetic variants that affect the volume of distinct parts of the brain [14–17].

In this review, we discuss the role of population-based neuroimaging studies in linking iron and other dietary factors to variations in brain integrity. We focus on iron, but briefly review other neuroimaging studies of diet and the brain. We also point out confounds and caveats to be aware of when interpreting these studies. Despite possible caveates, there has been progress in understanding molecular pathways that mediate dietary effects on the brain. Several genetic variants are associated with differences in iron metabolism, including its storage and transport; in fact, the most common genetic disorder in the world—*hemochromatosis*—is a disorder of iron metabolism, and affects 1 % of the population in some countries (e.g., Ireland). We describe current evidence relating iron levels to cognitive function, and new connections between brain integrity and genes that affect iron transport [18]. As brain databases expand to include ever-larger populations, neuroimaging can help to identify dietary factors and related genetic variants that consistently affect the brain, sometimes revealing mechanisms at the molecular level that mediate these effects.

## Brain imaging and diet

Magnetic resonance imaging (MRI) and its extensions allow us to observe, measure, and quantify neuroanatomical structures in a non-invasive fashion. When large numbers of individuals are scanned, it is possible to study patterns of brain abnormalities due to disease, and localize subtle anatomical differences that relate to variations in cognitive abilities, or even to single base pair variations in the genome, as we noted earlier [14]. Structures of interest (such as the volume of the hippocampus—a key structure for learning and memory) can be measured, based on individual brain scans. The relative size and integrity of anatomical structures can be compared across entire populations to identify factors that affect them, adversely or positively. Additionally, brain mapping studies allow, through careful co-registration, each individual subject’s brain scan to be mapped into a common 3D coordinate space, enabling inter-subject or inter-group comparisons. In this sense, subjects’ brain data can be compared at all 3D points—or voxels—of the image. These rapidly developing methods for image analysis have resulted in a range of brain-derived measures that can be analyzed statistically, to determine factors that affect brain structure and function, and factors that preserve and promote the integrity of the brain.

## Measuring brain integrity

Several different methods are used to image the brain. Each method is unique and can reveal various anatomical, functional, metabolic, or chemical attributes of the brain. Our focus in this review is on recent discoveries made with MRI and diffusion-weighted MRI (dMRI)—by far the most widely used methods for exploring the physical structure of the human brain, and factors that affect it. As noted earlier, standard anatomical MRI—the kind employed most widely in clinical assessments and research studies—is ideal for visualizing patterns and variations in brain structure, especially in the cortical gray matter. Diffusion MRI is more useful for mapping the integrity of white matter fiber tracts and their connectivity. Fractional anisotropy (FA) is one of the most widely accepted measures to represent white matter tissue integrity that is obtainable from DTI. FA, measured on a scale of 0–1, reflects how directionally constrained the diffusion of water is, along axons in a given volume such as a voxel. As a general rule of thumb, higher FA values may imply more coherent or intact axons, or higher degrees of myelination, while lower FA may reflect loss of integrity and white matter injury. Even so, there are known exceptions to this rule in highly convoluted regions of the white matter, because the FA measure appears to be reduced in regions where fibers cross each other, even when the integrity of the fibers is high.

**Fig. 1:**
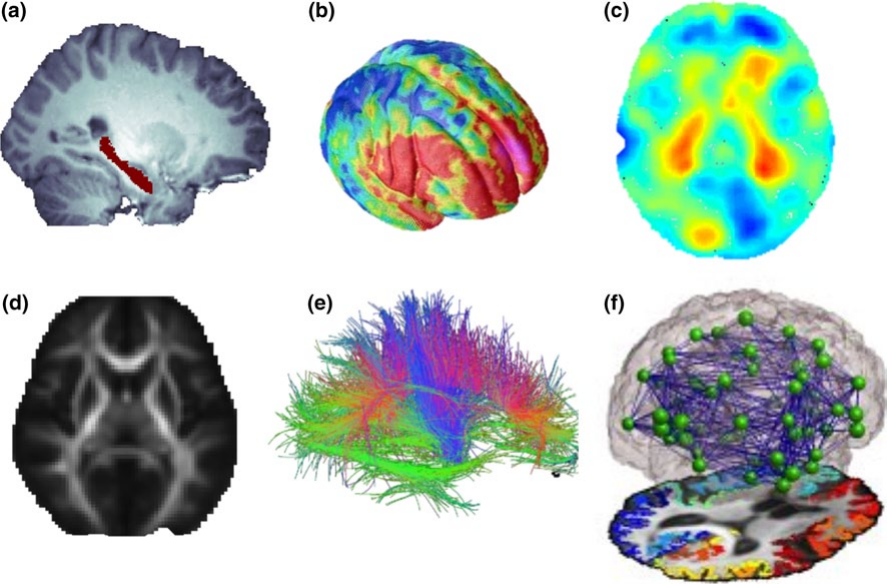
A variety of brain measures and features may be extracted from standard MRI and diffusion based MRI. When measured in a population of subjects, the observed variation in these brain traits can be studied in relation to genetic variation, and environmental influences such as education or diet, to identify factors that may promote or harm the brain. Population-based analyses include mapping out the overall degree of genetic influence or heritability for different brain measures, and effects of candidate gene or dietary or environmental factors. Features analyzed may include, but are not limited to: **a** volumes of brain structures such as the hippocampus; **b** cortical thickness or folding patterns; **c** regional brain volume differences with tensor based-morphometry maps; **d** maps of white matter integrity based on DTI, including measures of fiber coherence, such as fractional anisotropy (FA); **e** shapes and integrity of tracts of the brain; and **f** structural brain networks and the strength of cortical connections via white matter fibers, and topological measures based on such networks

Figure 1 shows various MRI-based features that may be extracted from standard MRI and dMRI techniques. Once these measures are derived, statistical methods from quantitative genetics and epidemiology may be used to map out effects of different dietary, genetic or other factors on these brain measures. Using a database of brain scans, one can study a variety of questions, such as those investigating

- the volume of specific structures in the brain, such as the hippocampus [14];
- cortical thickness or folding patterns [19, 20];
- regional brain volume differences, assessed using “tensor based-morphometry” maps (TBM; [21]);
- maps of white matter integrity such as diffusion tensor imaging (DTI)-based fractional anisotropy (FA) [22];
- the trajectories, shapes, and integrity of tracts of the brain [23]; and
- the structural human brain network—including the strength of cortical connections via white matter fibers and topological measures based on such networks [24–26].

Psychiatric disorders, such as schizophrenia and bipolar illness, neurological diseases such as Alzheimer’s disease, multiple sclerosis, and even viral infection with the human immunodeficiency virus (HIV) [27] lead to identifiable patterns of anatomical abnormalities and loss of tissue integrity, which are clearly seen in imaging studies. Remarkably, even a person’s diet can have a detectable influence on brain structure and function [28, 29]. Neuroimaging studies confirm that brain integrity is harmed by many drugs of abuse–ecstasy [30], methamphetamine [31, 32]—and excessive alcohol consumption [33]. Perhaps surprisingly, neuroimaging studies also have the power to detect the more subtle effects of specific daily nutrients. If properly designed (see caveats below), they can relate neurological changes and differences in brain integrity to specific dietary factors.

It has been shown, for example, that a group of pre-term infants fed a high-nutrient diet had significantly larger caudate volumes years later as adolescents [34] than those fed a standard diet; males in the high nutrient group of the study also had higher verbal IQ. Before describing more studies of dietary factors and their effects on the brain, we start by noting several major caveats that apply to many epidemiological studies of nutrition and diet, whether or not they use neuroimaging.

## Caveats in studying nutrition and the brain

First, in a purely observational study of any population, the intake of any one specific nutrient may be correlated with a host of other dietary factors or habits, making it difficult to isolate the effects of any one factor. However, more perniciously, our intake of any specific food or dietary component—such as sufficient iron or vitamins—may be statistically associated with better (or poorer) diet overall, socio-economic status, or with ethnic or demographic factors. The presence of these confounds makes it crucial, where possible, to control for effects of other possible determinants of brain function that may be driving the associations with a dietary factor.

For example, in studies of fish consumption, both geographical and cultural differences may affect how much fish there is in an individual’s diet (e.g., proximity to the coast). Economic factors are relevant as well. Household income may affect diet, and, depending on the population studied, socio-economic status may correlate with other health-related behaviors, for example with high or low body mass index—which is known to relate to brain structure.

Second, people who make healthy dietary choices tend to make health-promoting choices in other aspects of life, such as increased cardiovascular exercise. They may also avoid excessive alcohol intake or drug abuse, which are detrimental to the brain. Third, there are some associations between educational level and dietary choices, making it difficult to disentangle the primary effects of education on the brain, and effects of education on diet and food choices [35].

As such, it is vital to bear in mind that many studies of dietary factors and the brain are cross-sectional and do not employ a “double-blind” interventional design that introduces a nutritional factor in a randomized way. Such a design would be needed in a clinical trial to make causal statements about specific effects of the nutrient. As such, associations seen cross-sectionally need to be assessed critically. As we will see later, some genetic studies can get around this issue using the concept of “Mendelian randomization” [36]. First, however, we will review a number of studies tracking associations between dietary habits and measurable characteristics of the brain (Table 1). We note that a study not controlling for confounders is not necessarily invalid, as it may report a correlation that truly is present. Care is often needed in critically assessing what may be driving the association, what is the most likely cause, and how likely it is to generalize to new studies or individuals considering dietary changes.

Several studies focus on the consumption of fish, which varies widely across the world. Greater fish consumption is linked to better preservation of neuroanatomical integrity, including reduced white matter abnormalities in an elderly population [37, 38]. Specifically, levels of docosahexaenoic acid (DHA), a form of omega-3 fatty acid, measured from red blood cells, may preserve brain integrity. DHA levels have been associated with greater brain volumes and reduced white matter lesion burden in older, dementia-free adults [39].

Folate and B-vitamins, also sold widely as dietary supplements, show associations with cognitive performance in the elderly, in some but not all studies, as well as to differences in brain structure; vitamin B12 levels have been associated with differences in shapes of brain structures including the hippocampus and caudate [40] as well as gray matter volume in general [41], and white matter lesions [42].

Studies of healthy populations tend to be the best controlled for confounds, but unmodeled factors may drive some of the detected associations. As noted earlier, some cohorts include people who are depressed, or even mildly cognitively impaired, and run the risk of picking up an effect of illness on diet, as being ill may affect overall appetite and dietary choices. Chronic illness can affect body mass index and the ability to exercise or engage in any physical activity, and both affect brain structure and function [35]. People who are becoming ill may also change their diet in an effort to improve it. This can lead to paradoxical effects, whereby beneficial dietary changes may even appear to be associated with poor health or worse outcomes, if people are more inclined to pay attention to their diet when they are ill. Studies of vitamin intake in particular can be confounded by this illness effect.

Small amounts of choline, a nutrient also in the B-vitamin family, are created in our bodies, but the major contributor to our choline levels is our diet. Greater choline intake is associated with better cognitive outcomes in elderly and a reduced overall burden of white matter hyperintensities on MRI [43]. Elevated levels of the amino acid, homocysteine, which depend in turn on our intake of folate and B vitamins, can also affect brain integrity on MRI [44]. People with high homocysteine levels have an increased risk for stroke, Alzheimer’s disease and age-related memory impairment [45] through facilitated build-up of toxic beta–amyloid and tau in the brain [46, 47]. As those with high homocysteine levels have a profile of greater atrophy, early interventions through homocysteine lowering diets and drugs may be considered as means to resist brain atrophy in the elderly. In fact, recently, dietary supplements of B-vitamins, such as folic acid, have been reported to lower the risk for cognitive decline and AD, and they may work by lowering homocysteine levels [48].

**Table 1:** Nutrients that have been shown through neuroimaging techniques to be related to the integrity of the living human brain are listed, along with the specific associated structure from imaging. This list is not exhaustive, and several other studies not listed exist, particularly for iron measures. Despite these reported associations, the caveats listed in the main text apply to interpreting any causal connection between nutrition and the brain

Nutrient	Imaging modality	Specific structures	Study
Choline	MRI	White matter hyperintensity	[43]
Omega-3	MRI	ACC, hippocampus, amygdala	[49]
-DHA	MRI	Brain volume and white matter lesions	[39]
-EPA	MRI	Medial temporal lobe	[50]
Calcium	MRI	Brain lesion volume	[51]
Vitamin D	MRI	Brain lesion volume	[51]
Vitamin D	MRI	Brain parenchymal fraction	[52]
Vitamin D	MRI	White matter hyperintensity	[53]
Vitamin B6	MRI	Gray matter volume along the medial wall, anterior cingulate cortex, medial parietal cortex, middle temporal gyrus, and superior frontal gyrus	[41]
Vitamin B12	MRI	White matter lesions	[42]
Vitamin B12	MRI	Gray matter volume in superior parietal sulcus	[41]
Vitamin B12	MRI	Hippocampus and caudate	[40]
Brain iron	MRI *T*_2_*	Thalamus and basal ganglia–pallidum, putamen, and caudate nucleus	
Brain iron	MRI *T*_2_*	Thalamus	[54]
Brain iron	MRI-field-dependent R2 increase (FDRI)	Basal ganglia	[55]
Brain iron	MRI-susceptibility-weighted imaging	Putamen	[56]
Serum iron	MRI R2	Caudate nucleus, globus pallidus, corpus callosum	[57]
Serum transferrin (iron transport)	MRI and DTI	Ventricles, hippocampus, basal ganglia, external capsule, superior longitudinal fasciculus, cingulum	[58]
Serum transferrin	MRI R2	Caudate nucleus, globus pallidus, putamen, corpus callosum	[18], see Fig. 2
Choline	MRI	White matter hyperintensity	[58]

By far the most common nutritional disorder in the world is iron deficiency [59], which can adversely affect the developing brain (reviewed in [60]). Elevated iron levels can also promote degenerative disorders in the elderly [61]. The key need for iron homeostasis in the brain and body, and the serious adverse effects when iron levels depart from the normal range, have made it an interesting target of study.

## Iron, transferrin, and the human brain

Metal ions^1^ are increasingly important in helping to understand the development and progression of neurological diseases. As mentioned previously, copper, zinc and iron are all particularly critical for brain development. As with iron, inadequate concentrations of copper can cause anemia and developmental delays, while excess amounts of copper have been associated with neurodegenerative diseases such as Parkinson’s and Alzheimer’s diseases [64]. Additionally, zinc also plays a crucial role in neurodegeneration [65]. However, among the group of metal modifiers, the association of iron to the brain is the one of the most well studied, particularly in the context of additional genetic modifiers. Here, we briefly summarize known associations between brain measures and iron levels in the body (see also reviews by [2, 66–68]).

Iron-deficient diets are associated with poorer cognitive achievement in school-aged children [69]. In regions of the world where iron deficiency anemia is prevalent, such as East Africa, iron supplements can increase motor and language capabilities in children [70]. In children with ADHD, brain iron levels differ in thalamic regions [55]. While several of these studies use *T*_2_* imaging, exact quantification of iron content in the brain tissue is not readily possible in vivo. Indirect measurements and conclusions regarding iron content are obtained from of transverse relaxation times: these measure the decay of the magnetic signal due to perturbations in the magnetic field due to nearby “spins”; this rate of decay is generally different for various types of tissues. The “spin” is a property of the proton, which is used to create the nuclear magnetic resonance signal that we generate and measure with MRI. The decay of the MRI signal can be measured and modeled mathematically using a variety of “relaxation times”, which include *T*_2_*, *T*_2_, and *T*_2_’ [71]. These are all related by the following formula: $(T_{2} *)^{ - 1} = \,\left( {T_{2} } \right)^{ - 1} + \left( {T_{2} '} \right)^{ - 1}$. The relaxation time *T*_2_ is tissue-specific, whereas *T*_2_’ is associated with external field effects and includes all individual contributions from macroscopic and microscopic magnetic field inhomogeneities [72]. Therefore, the *T*_2_* relaxation time depends on the intrinsic *T*_2_ relaxation time as well as all individual macroscopic and microscopic magnetic field inhomogeneities. This measure is therefore affected by disturbances in the global and local magnetic field (i.e., inhomogeneities) as well as water diffusion in tissues, and in the presence of paramagnetic substances; these physical and biological considerations must be taken into account when interpreting results.

Iron deficiency can impair cognitive development, but iron overload also damages the brain. However, this damage is usually evident only later in life. Brain iron regulation is disrupted in several neurodegenerative diseases including Alzheimer’s disease [73], Parkinson’s disease [74], and Huntington’s disease [75], all of which involve abnormally high brain iron concentrations in neuroimaging studies. These high iron concentrations may cause neuronal death [76, 77].

Iron transport into the brain must be carefully regulated, as insufficient or excess iron can have devastating neurocognitive effects. Iron is transported into the brain, and throughout the rest of the body, by the iron-binding protein, transferrin, which regulates iron transport along with specialized transferrin receptors [78].

Transferrin levels can increase in iron-deficient states: when iron levels are low, the liver compensates by producing more transferrin [79], and less transferrin in cases of iron overload [80]. This iron level-dependent fluctuation in transferrin levels means that transferrin itself can serve as a good proxy measure of iron availability to the body. In fact, serum levels of iron fluctuate greatly [81] and depend on dietary factors such as vitamin C intake [82] and the time of blood collection [83]. The gold standard for measuring iron levels in the body is an invasive bone marrow or liver test, but these are not practical to use on volunteers in research studies. On the other hand, transferrin levels are easily measured with a blood test. This gives a reliable and reproducible index of the long-term availability of iron to the brain [84, 85].

**Fig. 2:**
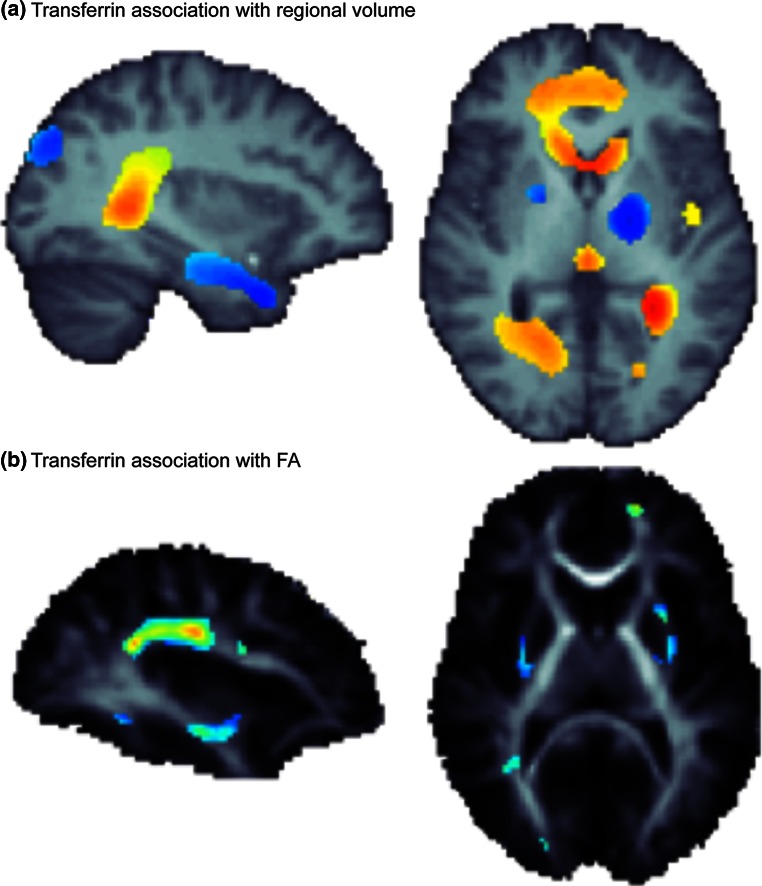
Using data presented in Jahanshad et al. [18], here we show the association of serum transferrin with **a** variations in local brain volume, including both positive associations (*blue*), and negative associations (*red/yellow*); and **b** variations in regional measures of fiber integrity as measured through diffusion based fractional anisotropy (FA). All FA associations were negative, such that as serum transferrin increased, regional integrity was reduced

Most of the brain’s iron is found in microglia and oligodendrocytes, where it supports myelination [67] and where iron homeostasis is maintained in the brain. Recently, we found that in healthy young adults, fractional anisotropy (FA), the most common measure of white matter integrity computed from DTI scans of the brain, is directly related to serum transferrin levels taken during adolescence [18]. Lower transferrin levels (indicating adequate to higher iron levels) reflected higher FA, or greater brain integrity (Fig. 2). This strong correlation suggests the importance of iron levels in the developing brain, and the ability for non-invasive brain imaging^2^ to trace these effects even in healthy young people.

## Neuroimaging genetics and iron-related genes

Iron enters the body mainly through the foods we consume. Even so, genetic factors affect the way our bodies absorb and process many nutrients, including iron, through various regulatory proteins involved in its metabolism.

Several genes have been linked to iron homeostasis, including: *ACO1, CALR, CD163, CP, CUBN, CYBRD1, DHCR7, EXOC6, FLVCR, FTH1, FTL, FXN, GAST, GSTP1, HAMP, HCP1, HEPH, HEPHL1, HFE, HFE2, HMOX1, HMOX2, HP, HPX, IREB2, PGRMC1, PGRMC2, SLC11A2, SLC25A37, SLC40A1, STEAP3, TF, TFR2, TFRC, TNF* [87]. Some of these show associations in multiple ethnic groups [88]. However, not all have been shown to have neurological implications.

Table 2 summarizes some of the genes involved in iron homeostasis that have so far been studied with respect to human brain structure, cognition, or neurological diseases.

**Table 2:** Iron-related genes included in  studies attempting to associate common variants (SNPs), copy number variants, or expression of these genes to neurological diseases and traits including neurodegenerative and neuropsychiatric diseases, behavior disorders, brain structure, and cognitive measures are listed

Gene	Association	Study	Significant association found?
*HAMP*	Brain tumors	[89]	+
*ACO1*	Brain atrophy	[90]	+
*HFE*	AD	[91]	+
	AD	[92]	Some
	AD	[93]	None
	AD	[94]	None
	ALS	[95]	+
	ALS	[96]	+
	ALS	[97]	None
	ALS	[98]	None
	Brain integrity (DTI-FA)	[18]	+
	Brain tumors	[89]	+
	MS	[99]	+
	PD	[93]	+
	PD	[100]	None
	RLS	[101]	None
	Autism	[102]	+
	Autism	[103]	None
	In-vivo brain iron	[104]	+
*HFE2*	Brain tumors	[89]	None
*HFE* + *TF*	Memory	[105]	+
	AD	[106]	+
*FTL*	Neuroferritinopathy	[107]	+
*NEO1*	Brain tumors	[89]	+
*TF*	AD	[108]	+
	AD	[109]	+
	AD	[110]	None
	AD	[111]	None
	AD	[92]	None
	PD	[100]	+
	PD	[112]	None
	SZ	[113]	+
	SZ	[114]	None
	Brain integrity (DTI-FA)	[18]	None
*TFR2*	Brain tumors	[89]	+
*TFRC*	Brain tumors	[89]	+
*FPN1*	MS	[99]	+
*HEPH*	MS	[99]	+

(*AD* Alzheimer’s disease, *PD* Parkinson’s disease, *MS* Multiple sclerosis, *RLS* Restless leg syndrome, *ALS* Amyotrophic lateral sclerosis, *SZ* schizophrenia)

The genes presented in the table include *HFE, HFE2, HAMP, TFR2, ACO1, FTL, NEO1, TFRC, FPN1, HEPH* and *TF*. Approximately 80 % of hereditary hemochromatosis (HH) is explained by mutations in the *HFE* gene (or Type 1 HH), but other genes including *HFE2*, *HAMP, TFR2* and *FPN*-related genes are all involved in non-*HFE* HH through their direct or indirect regulation of hepcidin, an iron-regulating hormone in the liver [115, 116]. *HFE2* is associated with Type 2A juvenile hemochromatosis (JH)-a rare autosomal recessive form of hemochromatosis that causes severe organ damage and premature death before age 40. The non-*HFE* HH gene, *HAMP*, is also known to be associated with Type 2B JH; *TFR2*, expressed almost entirely in the liver, also is involved with hepcidin synthesis and can lead to a form of iron overload that is similar to *HFE* HH, also without *HFE* involvement; this is known as Type 3 HH [115]. *FPN1,* or *SLC40A1*, codes for the ferroportin protein that transports iron from the inside to the outside of a cell. It is essential in development, and is associated with Type 4 HH, or ferroportin disease, an autosomal dominant disease [115, 116].

*ACO1,* also known as iron regulatory element binding protein 1, binds to ferritin and transferrin mRNA; polymorphisms in the gene have also been associated with age-related macular degeneration [117].

Variants in the *FTL, NEO1, TFRC*, and *HEPH* genes alone are not causes for HH, but these genes are essential in regulating iron homeostasis and transport. *FTL* is the gene encoding the ferritin light polypeptide, one of the two subunits of the ferritin molecule; it is another gene important for iron homeostasis. Mutations in the gene were first used to describe neuroferritinopathy, evident from iron deposition in the basal ganglia [107]. *NEO1,* is closely related to a tumor suppressor gene *DCC* and binds *HFE2*, and through their interaction, regulates iron homeostasis in hepatocytes and possible skeletal tissue [118, 119]. *TFRC* encodes the transferrin receptor protein 1 (TfR1)—the protein required for the uptake of transferrin-bound iron in human cells [120]. *HEPH* or hephaestin, is necessary for transporting iron out of the small intestine’s enterocytes and into the circulation [121].

Some of these studies found no association between the specific candidate genes and the disease of interest, leading to inconsistent evidence, especially for the transferrin gene, *TF*. Commonly-carried variants in *TF* (in addition to variants in *HFE*) accounted for ~40 % of the variance in serum transferrin levels in two normal, healthy populations of approximately 400 individuals [122]. Variants in the *TF* gene were associated with schizophrenia in a Chinese population of approximately 300 cases and 300 controls [113] but not in a Japanese population with approximately the same number of individuals [114]. Also, comparing about 200 cases to 200 controls, [100] found *TF* polymorphism G258S was associated with PD, but this effect was not replicated in a Spanish population with roughly the same number of patients [112].

Additionally, the *HFE* or *hemochromatosis* gene is associated with iron metabolism disorders. The rarer C282Y mutation in the gene can cause hereditary hemochromatosis (a genetic disorder of iron overload), while the more common H63D variant has been the repeated focus of attention for degenerative brain diseases [123]. Copy number variants in the gene may also be over-represented in people with autism spectrum disorder [102]. Variants in iron-metabolism genes clearly play a role in brain development and degeneration, and this has led to efforts to find molecular pathways by which these genes affect the brain. Brain imaging can facilitate the search for these molecular pathways—by measuring the degree to which these genetic risk factors are associated with brain differences.

**Fig. 3:**
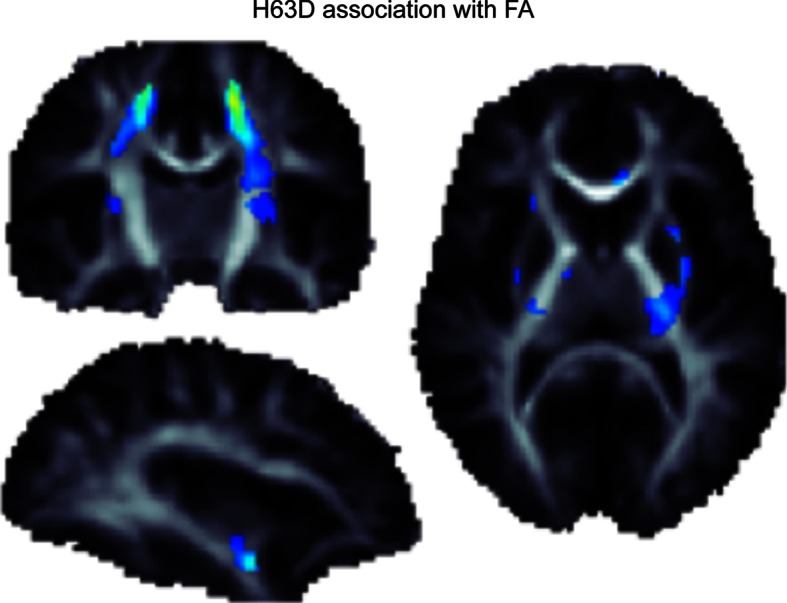
Using data presented in Jahanshad et al. [18], we show the association of the H63D polymorphism in the *HFE* gene with variations in localized fiber integrity, as measured through diffusion-based fractional anisotropy. All FA associations were positive, so the minor allele at H63D showed increases in FA in a dose-dependent manner

For example, we recently used a study design involving twins—called a *cross*-*twin cross*-*trait* study—to show that commonly carried genetic variations contribute to the normal variation in both transferrin levels and brain integrity as measured with DTI scans In other words, there is *pleiotropy*—the same genes are implicated in both measures. When individually evaluating all common genetic variants in *TF* and *HFE*, variants that together explain ~40 % of the variation in transferrin levels [122], we found that healthy carriers of the H63D polymorphism in the *HFE* gene have characteristic differences in brain structure, Fig. 3.

This *HFE* variant is commonly associated with neurodegeneration later in life, but our study of younger people showed higher integrity in healthy carriers of the *HFE* variant, possibly due to better myelination, approximately 50 years before the average onset age of dementia. However, when examining the same variant in elderly men, Bartzokis et al. [105] found that the presence of the H63D *HFE* variant (and/or C2 allele of the transferrin gene) was associated with increased basal ganglia iron concentrations compared to noncarriers, suggesting iron overload in the brains of these carriers—a potential risk for degeneration and dementia. Many studies have addressed the association of the H63D variant and the *HFE* gene on Alzheimer’s disease, with mixed findings [123] regarding the direction of association, the interaction of genes, and the sex most affected. Brain imaging in larger cohorts may help to disentangle the effects of *HFE* and other iron-related genes on brain structure throughout life.

## Conclusion

Neuroimaging genetics now offers great power to understand genetic modulators of dietary influences and their metabolic pathways. By screening brain databases, we may be able to discover nutrients and related genes impacting the brain, deepening our understanding of how certain genes contribute to cognitive outcomes. Additionally, monitoring effects of these mutations with respect to diet as we age may reveal critical periods in the human lifespan when risk for disease is greatest, or when dietary effects are most pronounced. In people at high risk for iron overload disorders, for example, simple preventative nutritional monitoring can help delay, slow down, or even prevent cases of disease.

As we have noted, there are limitations in studies that use brain imaging to map nutritional or genetic effects on the brain. Despite the promise and growth of the field, replication in large samples is needed to boost confidence in the findings. As most single-site imaging studies are small and relatively underpowered to pick up dietary effects or genetic associations, large consortium efforts (e.g., ENIGMA; http://enigma.loni.ucla.edu) are underway to improve power in discovery and replication of subtle effects on the brain. Any actionable factor discovered to resist brain aging or promote brain integrity could impact the lives of hundreds of millions of people worldwide. Many large epidemiological studies now include brain imaging as part of our armory of tools to facilitate this quest.

## Abbreviations

MRI: Magnetic resonance imaging
dMRI: Diffusion magnetic resonance imaging
DTI: Diffusion tensor imaging
SNP: Single-nucleotide polymorphism
FA: Fractional anisotropy
TBM: Tensor-based morphometry
DHA: Docosahexaenoic acid
EPA: Eicosapentaenoic acid
AD: Alzheimer’s disease
PD: Parkinson’s disease
MS: Multiple sclerosis
RLS: Restless leg syndrome
ALS: Amyotrophic lateral sclerosis
SZ: Schizophrenia
ADHD: Attention deficit hyperactivity disorder

## Footnotes

1 At the physiological concentrations present in the brain, copper levels do not appear to influence the signal on standard brain MRI. However, paramagnetic materials such as copper (II) can be and are often used as contrast agents in MRI scans. A newer MRI imaging method, susceptibility-weighted imaging, takes advantage of the distortions in the magnetic field from susceptibility differences in tissue types due to paramagnetic or diamagnetic compounds, to map out cerebrovascular changes in the brain. While this is out of the scope of our current review, we point the reader to the following reference for more information [62, 63].

2 Non-invasive imaging procedures using MRI have also been developed to map iron concentrations in the brain. *T*_2_* contrast imaging [86] may be used to measure the degree of iron in living tissue. *T*_2_*, and its inverse, *R*_2_, are decay constants that measure of how fast the MRI signal decays, due to magnetic resonance relaxation phenomena. The physics of this process is quite well understood, and iron disrupts the process. As a result, the *R*_2_ relaxation parameter is increased by elevated iron levels in brain tissue. As such, *R*_2_ offers a relative, but not absolute, measure of brain iron levels. Another form of MRI imaging, known as field-dependent *R*_2_ increase (FDRI), uses field-dependent properties of tissues to quantify tissue iron [73]. In vivo evaluation of brain iron in Alzheimer's disease and normal subjects is also possible using MRI. This method may involve scanning a person on two different MRI scanners with different field strengths.
